# Retraction: Long non-coding RNA FENDRR inhibits migration and invasion of cutaneous malignant melanoma cells

**DOI:** 10.1042/BSR-2019-1194_RET

**Published:** 2023-12-22

**Keywords:** FENDRR, invasion, Malignant melanoma, migration

This article is being retracted from *Bioscience Reports* at the request of the Editor-in-Chief and the Editorial Board following receipt of a notification from a reader, alerting the Editorial Board to sections of the images in Figure 3A and 3B that are duplicated, both within these figures as well as in unrelated articles published elsewhere.

The Editorial Office has contacted the authors regarding the issues raised and the authors have not responded to the Journal's queries or the concerns raised. Given the extent of the issues raised, the Editorial Board stand by the decision to retract the article.

